# Behavior of Proteins under Pressure from Experimental Pressure-Dependent Structures

**DOI:** 10.1021/acs.jpcb.1c03313

**Published:** 2021-06-08

**Authors:** Beatriz Fernández del Río, Antonio Rey

**Affiliations:** Departamento de Química Física, Facultad de Ciencias Químicas, Universidad Complutense de Madrid, E-28040 Madrid, Spain

## Abstract

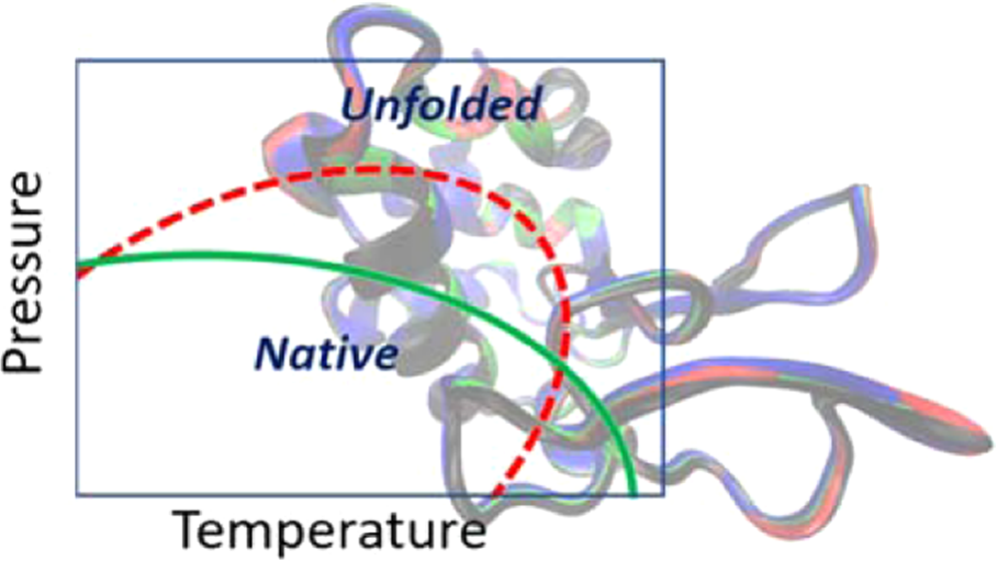

Structure-based models are coarse-grained representations of the interactions responsible for the protein folding process. In their simplest form, they use only the native contact map of a given protein to predict the main features of its folding process by computer simulation. Given their limitations, these models are frequently complemented with sequence-dependent contributions or additional information. Specifically, to analyze the effect of pressure on the folding/unfolding transition, special forms of these interaction potentials are employed, which may a priori determine the outcome of the simulations. In this work, we have tried to keep the original simplicity of structure-based models. Therefore, we have used folded structures that have been experimentally determined at different pressures to define native contact maps and thus interactions dependent on pressure. Despite the apparently tiny structural differences induced by pressure, our simulation results provide different thermodynamic and kinetic behaviors, which roughly correspond to experimental observations (when there is a possible comparison) of two proteins used as benchmarks, hen egg-white lysozyme and dihydrofolate reductase. Therefore, this work shows the feasibility of using experimental native structures at different pressures to analyze the global effects of this physical property on the protein folding process.

## I Introduction

Protein folding is a highly complex problem from a physical point of view. It involves many degrees of freedom, considering the number of atoms comprising both the polypeptide chain and the surrounding aqueous medium. In addition, from a dynamic point of view, it spans many temporal orders of magnitude. Therefore, a thorough description of the full process at the atomic level requires vast computational resources, is limited to small proteins, and to fast-folding processes.^1^

Many simplified, or coarse-grained, models have been developed to reduce the complexity of this problem from a computational point of view. They have been recently reviewed in the literature.^2,3^ By often using a simplified representation of the protein chain, a continuous description of the solvent, and different kinds of simplified interaction potentials, they provide a reasonable representation of the conformational space available to the chain under different conditions, at the cost of losing the detailed, microscopic information, which may result critical in certain occasions.^3−5^

Among the different interaction potentials developed, structure-based (or Go-type) interaction models have been widely used.^6^ They are based on the minimal frustration theoretical framework for protein folding,^7−9^ which proposes a funnel-shaped energy landscape, where the folded state occupies the energy minimum. Therefore, the stabilizing interactions of these models are defined from the inter-residue contacts present in the native state, experimentally determined. These models, thus, are used to analyze the thermodynamic and kinetic characteristics of the folding pathways, with surprisingly good results on many occasions.^10−14^

In the original definitions of these models, the effect of the protein sequence is completely ignored, which could in principle preclude the possibility of analyzing different interesting evidences such as the effect of mutations, the prediction of the native structure, or the change in some environmental conditions (ionic strength, pH, etc.), just to mention a few. Consequently, structure-based models have been enriched with many modifications that decorate the interactions having the sequence into consideration, and/or explicitly adding non-native interactions of different nature.^7,15−17^

These models have also been extended to account for the effect of pressure in the folding equilibrium between the native and the unfolded states. It has long been known that pressure unfolds proteins in a different way to the effect of temperature or chemical denaturants.^18−26^ The most accepted view assumes that water molecules are injected at high pressure (of the order of 3–5 kbar) into the cavities or void volumes existing in folded proteins, as a result of the incomplete packing of the hydrophobic cores.^27^ This fact produces unfolded states that are swollen versions of the native conformations. Moderate pressures also help reduce the cavities that are buried in the protein core in the folded structure, thus modifying the pathways and speeds of the folding processes.^28^ At a different level, pressure modifies the hydrophobic interactions, at least when they are described as a potential of mean force between hydrophobic groups surrounded by water.^29^^30^ As a matter of fact, this has been the basis of some coarse-grained models, which take pressure into account in the study of the folding/unfolding transition, as described below.

**Figure 1:**
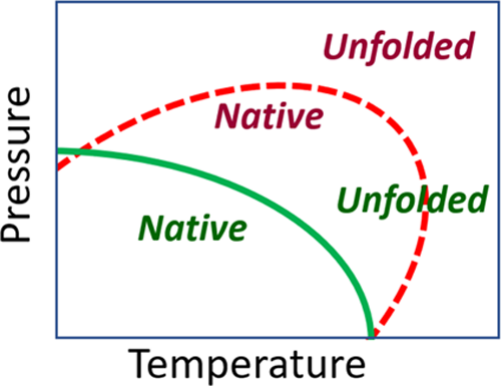
Schematic representation of the elliptic pressure–temperature phase diagram for a protein, showing two possibilities for the equilibrium line between the native (folded) and the unfolded states, with the same transition temperature at room pressure (bottom axis). The native state lies inside the corresponding elliptical region, while the outer area corresponds to the unfolded state.

This situation has been explained in thermodynamic terms by an elliptical phase diagram for protein stability in the pressure–temperature landscape, as sketched in Figure 1, which also shows the cold denaturation of proteins. Within this scenario, the effect of moderate pressures at room temperature and above can be different depending on the elliptical shape and orientation. If the phase diagram approximately corresponds to the green solid curve in Figure 1 (apparently, the most common situation), an increase in pressure above room conditions (the horizontal axis in the diagram) will destabilize the folded state, therefore, reducing its transition temperature. On the other hand, if the diagram is like that in the red dashed curve of Figure 1, an initial moderate increase in pressure will shift the equilibrium unfolding temperature toward larger values, thus stabilizing the native state. Which of these possibilities appears has been mainly assigned, following the Clapeyron equation for phase changes, to the sign of the difference between the partial molar volumes of the native and the unfolded states.^27,28^

Structure-based models have tackled pressure effects by defining interaction potentials for the native contacts with two attractive minima, separated by a desolvation barrier.^30,31^ The pressure scale modifies the relative depth of both minima, as well as the height of the barrier in between. Two different approaches have been proposed for this variation, and their effects have been very recently analyzed.^32^ The main conclusion from this study is that the interaction potential used determines whether moderate pressures either stabilize or destabilize the folded state, by shifting the melting equilibrium temperature toward higher or lower values, respectively.^32^ In our group, we have also checked one of these possibilities in a model similar to the one employed in this work.^33^

Interestingly, the effect of pressure in all of the studies mentioned above with structure-based models relies on a set of native contacts experimentally determined at room pressure, i.e., in one native structure alone. Proteins are considered quite compact molecules, and the effect of pressure on the native state (up to values where it is still stable) is considered only marginal. This has been experimentally proved in recent years when structures determined at high pressures have been reported. The structural deviations induced by pressures of several kbar produce root-mean-square deviation (RMSD) values of less than 1 Å with respect to the structure at 1 bar (see below), which could in principle be considered as a negligible change, even below the level of resolution of many coarse-grained models. However, these minor changes are not uniformly distributed along the structure and, therefore, can have an influence even in the behavior of these reduced models.

Given this situation, we propose in this work to use a very simple structure-based model in which the pressure effects are not included in the mathematical description of the interaction potential, but in the native structure itself, therefore recovering the original ideas underlying these models:^8,34,35^ the native structure mainly determines the full shape of the energetic folding landscape. If pressure affects the native structure, it must affect the folding landscape as well, and that is what we want to check with our results in this work.

Obviously, the model is incomplete in several senses, including neglecting an explicit representation of the solvent, and therefore of the possible effects of pressure on it.^36^ This also precludes the possibility to study the cold denaturation process.^37^ Our idea is to check whether the apparent minute changes in the native structure created by pressure are able to modulate the thermodynamic and kinetic characteristics of the folding transition studied with a coarse-grained model. This is relevant since the model resolution is, at much, of the same level as the pressure-induced structural changes themselves. As we will show, the answer to this challenge is quite promising.

## II Methods: Simulation and Interaction Model

To keep the coarse-grained model as simple as possible, in this work, we use our previous expertise by employing a Monte Carlo sampling method on a linear chain composed of *N* beads, each one representing a single amino acid.^38,39^ To avoid sampling getting trapped in local minima, in simulations devoted to thermodynamic properties, we add a replica exchange strategy,^40,41^ where different replicas are simulated at different temperatures (parallel tempering). The Monte Carlo sampling includes both local movement (single-bead) trials, where a single unit is rotated preserving the connectivity with its neighbors along the polypeptide sequence, and collective (multi-bead) movement trials, where one bead randomly chosen in the chain suffers one of the local movements, but the rest of the chain, from the next bead to the end, is parallelly shifted to a new position, preserving the vectors between neighboring units. This way, we generate configurations of the system that correspond to the conformational equilibrium distribution of the protein model at each of the temperatures included in the parallel tempering procedure. The method has been thoroughly described in previous works.^38,39^

As it corresponds to a structure-based model, the interactions are defined from the native conformation, as taken from the experimentally solved structures available in the Protein Data Bank (PDB).^42^ We then select the contacts present in the native conformation at a given pressure and use an energy term defined as a truncated harmonic well centered at the native distance and whose depth, equal for all of the native contacts, defines the energy unit of the model.^38^ Its mathematical definition is
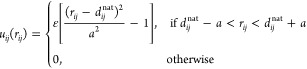
1

In eq 1, *r*_*ij*_ is the distance between the beads representing residues *i* and *j*, *d*_*ij*_^nat^ is the corresponding distance between their α-carbons in the native state; ε = 1 defines the energy unit for the model, and *a* indicates the width of the attractive well for the native contacts. Values of *a* = 0.5 or 0.6 Å have provided correct results for different proteins in previous works from our group.^13,43,44^ Here, we use *a* = 0.6 Å. The reduced energy scale imposed by the ε value mentioned above implies a reduced temperature scale as well. All of the reduced (dimensionless) thermodynamic magnitudes are denoted with an asterisk in Section IV.

In addition to the attractive interaction potential for the native contacts, the model includes an excluded volume, hard-sphere repulsive potential acting among any pair of model units.^38,39^ This term avoids the overlapping of the model beads.

As explained in Section I, the influence of pressure in the folding process is considered only from its effects on the native conformation. As shown in the next section, pressure modifies the number and position of some native contacts, which are passed to eq 1 to create our model interactions, which become this way “pressure-dependent”. Thus, our pressure scale corresponds to that reported for the experimental structures used as input.

The thermodynamic results presented here correspond to an average of 10 independent runs in every case. In each run, we use 50–60 temperatures in the parallel tempering scheme, depending on the complexity of the folding process found for every protein reported, to warrant a proper traveling of the replicas along the full set of temperatures. At every temperature, 3 × 10^6^ Monte Carlo cycles (each one involving *N* trial conformations) are sampled for thermalization, and 10^7^ additional Monte Carlo cycles are computed for data recording and analysis. The statistical reproducibility of the results from the 10 independent runs computed from every contact map guarantees the choice of temperatures and the statistical quality of the sampling.

The folding kinetics calculations use the same interaction scheme but a different sampling procedure, which we have also previously used in our group.^45,46^ Many independent Monte Carlo simulations at a single temperature (our approximation to room temperature, *T*_r_) are started from different conformations belonging to the unfolded state (taken, as a matter of fact, from the high-temperature replicas of the thermodynamic procedure). The simulation continues with only local moves (single-unit moves, as described above) until a certain “folding criterium” is fulfilled. Here, we use an RMSD threshold value as a stop condition (computed between every sampled conformation and the experimental native structure, at the level of α-carbons). To determine this threshold, the RMSD distribution of the conformations sampled at *T*_r_ in equilibrium (parallel tempering) simulations is computed. The RMSD value that frames 90% of the conformations at that temperature is chosen as a folding criterion. This is usually around 1 Å. The number of Monte Carlo cycles needed to reach that value from the unfolded state is recorded. This way, the evolution of a population of unfolded conformations at room temperature is quantified as a function of the number of MC cycles, which is considered to be proportional to a real time scale. In this work, 10^4^ folding trajectories are computed for the calculation of the folding kinetics for each protein at a given pressure. Our estimation for the room temperature is *T*_r_ ≅ 0.90 *T*_m_, where *T*_m_ is the equilibrium temperature for the folding/unfolding transition, which we get from the analysis of the parallel tempering equilibrium simulations.

## III Proteins Considered

If one analyzes the structures currently present in the PDB^42^ (March 2021), just a handful of proteins solved by solution NMR at different pressures (up to about 3 kbar) can be found. On the other hand, around one hundred structures solved by X-ray diffraction have been deposited. Many of them correspond to moderate pressures (several tens of bar), but a few of them rise up to 9 kbar, a pressure that should correspond to the pressure-unfolded state according to the current knowledge.^19,47−51^ It may seem that the crystal structure somehow dampens the real pressure felt by the protein molecule, a fact that was already noticed from the very first solved X-ray protein structures at high pressures.^52^ Nevertheless, this may also imply that the pressure felt by the protein is not exactly the same as the pressure exerted on the crystal inside the experimental setup. Therefore, some caution should be possibly taken about the quantitative values of the pressures reported.

As it could be expected, some of the proteins whose structures have been solved at high pressures correspond to molecules whose folding behavior has been well characterized at room conditions. Therefore, we have chosen the X-ray structures of hen egg-white lysozyme (HEWL)^53^ and *Escherichia coli* dihydrofolate reductase (DHFR).^54^Table 1 presents the relevant information about the native structures considered for this work. Pressure makes structures slightly more compact, as shown by a decrease in the radius of gyration, *R*_g_. The effect, however, is very small, with even some deviations from this trend in certain cases, as it happens with the two highest pressures in DHFR. From the structural similarity level, the RMSD values are in all of the cases well below 1 Å when high-pressure structures are compared with the same protein at atmospheric pressure.

**Table 1:** Relevant Data of the Structures Taken from the PDB^42^ to Represent the Effect of Pressure on Hen Egg-White Lysozyme (HEWL) and Dihydrofolate Reductase (DHFR)

protein	pressure (kbar)	PDB file	*R*_g_ (Å) (Cα only)	RMSD (Å) to 1 bar (Cα only)	number of native tertiary contacts^a^
HEWL^53^*N* = 129	10^–3^ (room pressure)	4WLD	13.77		292
	1.9	4WLT	13.67	0.149	302
	3.8	4WLY	13.60	0.229	304
	6.0	4WM2	13.51	0.333	309
DHFR^54^*N* = 160	10^–3^ (room pressure)	5Z6F	15.22		376
	2.2	5Z6J	15.11	0.277	386
	4.0	5Z6K	15.04	0.339	385
	8.0	5Z6M	15.08	0.730	385

a “Tertiary contacts” in the last column amounts for native contacts present between residues *i* and *j* with |*i* – *j*| ≥ 4, excluding virtual bond angles and virtual torsion angles in the model.

**Figure 2:**
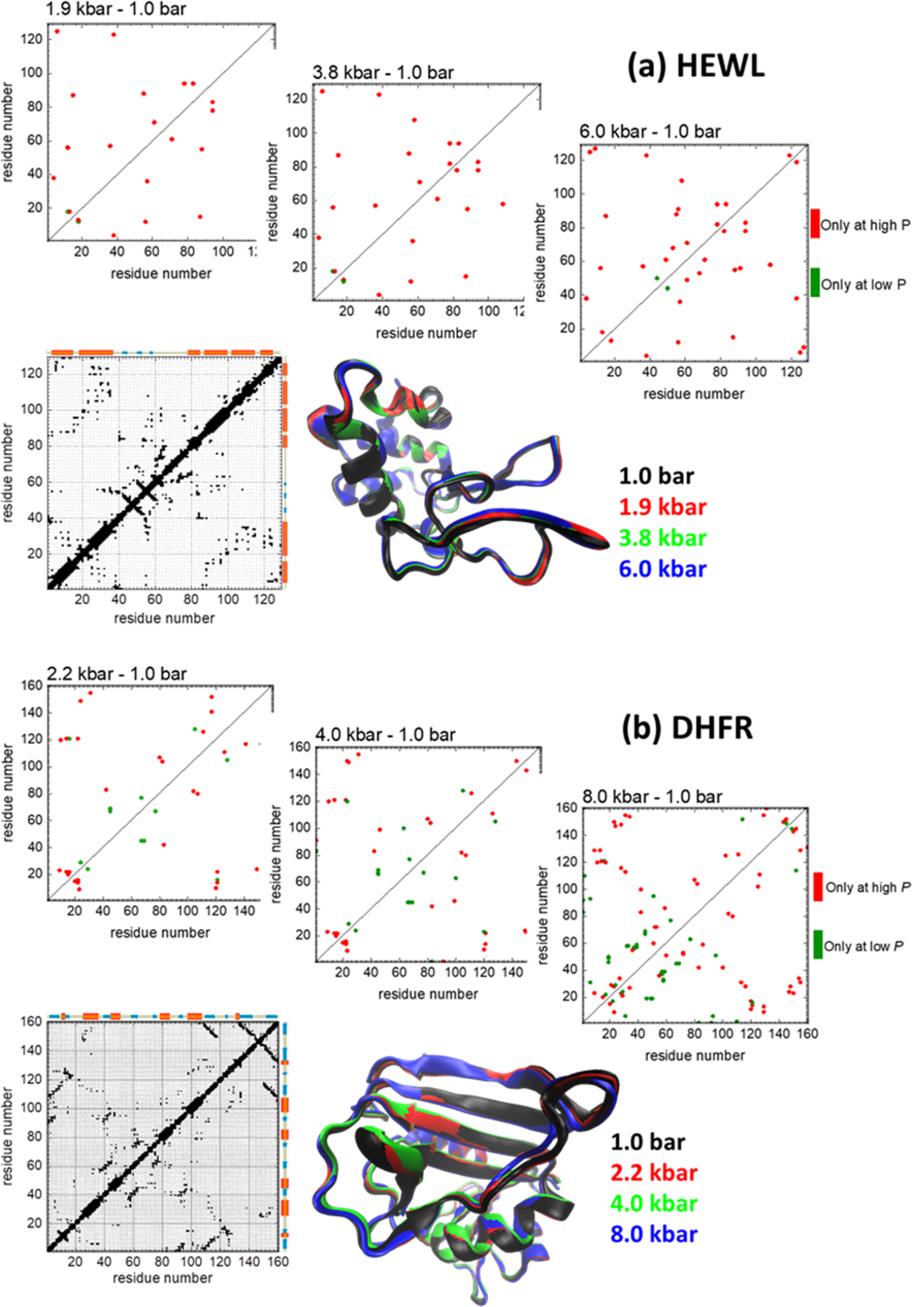
Difference contact maps (native contacts present at high pressure minus native contacts present at room pressure) for (a) lysozyme, HEWL, and (b) DHFR. The native contact maps for both proteins at room pressure are also shown, with a sketch of the elements of the secondary structure along the axes (orange: α-helices; cyan: β-strands). The ribbon diagrams show a superposition of the experimental native structures corresponding to the different pressures of each protein, color-coded as indicated.

The native contact maps, which define the attractive interactions in our simulation model, have been computed from the atomic coordinates and are depicted in Figure 2. They use a standard cutoff value of 4.5 Å between non-hydrogen atoms belonging to different residues to decide whether a contact is present or not in the native conformation at every pressure. In Figure 2, we also show ribbon diagrams with the superposition of the structures for both proteins at four different pressures, the native contact maps at atmospheric pressure, and the difference between the contacts present at high pressure with respect to the same protein at room pressure. In these plots, the first interesting differences between the two proteins considered in this work arise. Although HEWL essentially shows a monotonous increase in the number of native contacts as pressure increases, as it can be seen both in Table 1 and in Figure 2a, DHFR presents a more complex behavior, keeping an approximately constant number of contacts at the highest pressures (Table 1). As seen in Figure 2b, this is because native contacts both appear and disappear when pressure increases with respect to 1 bar contacts. Therefore, in HEWL, the increase in contacts can be assigned to a small reduction of the volume of cavities in the native state, as shown in the experimental results,^53^ which similarly affects different parts of the structure, since the new contacts are scattered over different sections of the contact map. In DHFR, on the other hand, pressure creates distortions in the structure, which generate new contacts in some regions but make others present at room pressure vanish, due to some water molecules being injected into the structure at moderate and high pressures.^54^

To properly discuss the results of these two proteins, there are some cautions we should remark. Given the simplicity in the coarse-grained representation of both the polypeptide chain geometry and the system interactions, a direct comparison with experimental results^55−64^ is not always possible. For example, HEWL presents four disulfide bonds in its native structure, which are preserved in the denatured state in many (fortunately, not all) thermal and pressure unfolding experiments under oxidizing conditions. In our model, these bonds are considered as any other native contact and, therefore, are prone to disappear along the unfolding process; therefore, we can only compare our results to experiments where the sulfur bridges are lost upon unfolding. In addition, the crystal structures of DHFR we are using are complexed with folate and NADP as ligands. Neither of them is considered in this work, where only the protein residues are accounted for.

## IV Results

### IV.I Thermodynamic Simulations

From the energy distributions obtained at each temperature, we can readily calculate the heat capacity curves. In the reduced units used in this work,
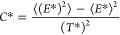
2

**Figure 3:**
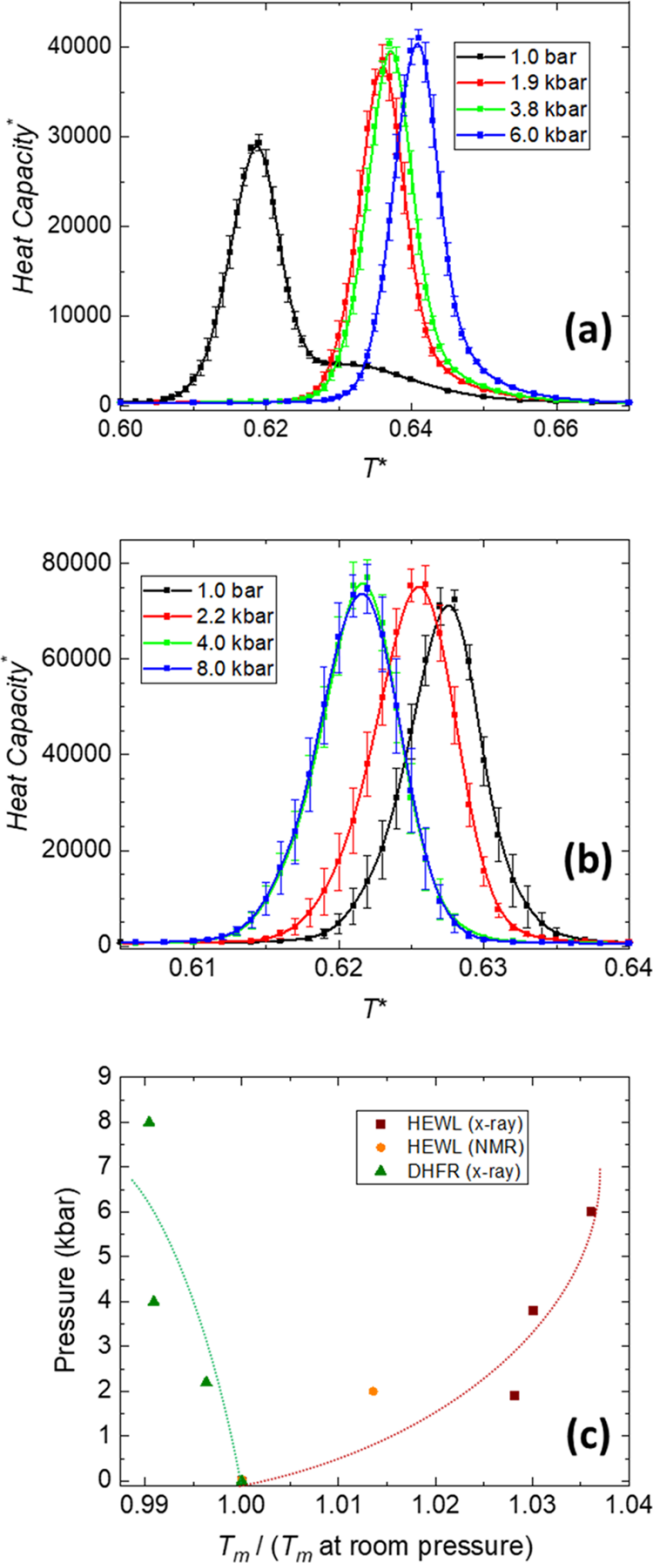
Simulation results for the reduced heat capacity against the reduced temperature for (a) HEWL and (b) DHFR, at different pressures. (c) Phase diagram representing the equilibrium transition temperatures for the different pressures, for both proteins. The statistical bars for these temperatures roughly correspond to the symbol sizes. The lines are just eye guides showing the global trends.

They are shown in Figure 3a for HEWL and Figure 3b for DHFR, for the four pressures used in each protein. The error bars included in these graphs are statistical errors of the mean values computed from the analysis of the 10 independent simulations run in every case. In Figure 3c, we summarize the results for the transition temperatures, *T*_m_, which are the absolute maxima (the peak temperatures) in the heat capacity curves, and correspond to the thermodynamic equilibrium between the folded and unfolded states. To better quantify the effects of pressure, they are normalized in Figure 3c with the value of *T*_m_ at 1 bar for the corresponding protein. As can be seen, both proteins behave in a different way: while for HEWL the equilibrium temperature is shifted toward larger values as pressure increases, the opposite situation appears for DHFR. In addition, HEWL at 1 bar shows a second transition at temperatures higher than *T*_m_, whose influence along the global thermal transition seems to be highly reduced when pressure increases, since only a minor asymmetry in the heat capacity curves is observed at high pressures. We shall further comment on this result below.

In Figure 3c, we have also included the results of HEWL computed from two NMR structures taken from the PDB: 1GXV (measured at 30 bar) and 1GXX (measured at 2 kbar), which represented the first solution structures of globular proteins under pressure.^65^ The first conformer in the deposited set has been used in each case to define the contact map for our simulation model, although all of the experimental conformers at each pressure are nearly identical to one another and provide the same behavior.^66,67^ Interestingly, the NMR results show the same trend as the X-ray ones, namely, a small stabilization of the native state as pressure increases. The quantitative results are not identical, though, probably reflecting the different pressure scales felt by the protein in solution and in the crystal structure, as commented on above. Anyhow, the results obtained for lysozyme are qualitatively coincident with the experimental evidence, showing an increase in the thermal stability of the native structure as pressure increases up to moderate values,^68,69^ related to the compression of internal cavities described for native structures.^53^ The experimental results, however, are highly dependent on pH and other factors,^69^ which cannot be taken into account in our coarse-grained model. This temperature–pressure behavior approximately corresponds to the equilibrium red dashed line sketched in the phase diagram of Figure 1.

DHFR, on the other hand, shows the most common behavior, with the equilibrium temperature shifted toward lower values as pressure increases, a tendency that is equivalent to the green solid line in the phase diagram of Figure 1. In this protein, the injection of water molecules inside the protein structure as pressure increases from room values to 4 kbar must be responsible for this behavior. Beyond that pressure, the protein core cannot accommodate more water molecules,^54^ and the result we obtain for the equilibrium temperature at 8 kbar is almost the same as that obtained at 4 kbar (see also the change in the trend shown for the radius of gyration, *R*_g_, of the native structures between these two pressures in Table 1).

**Figure 4:**
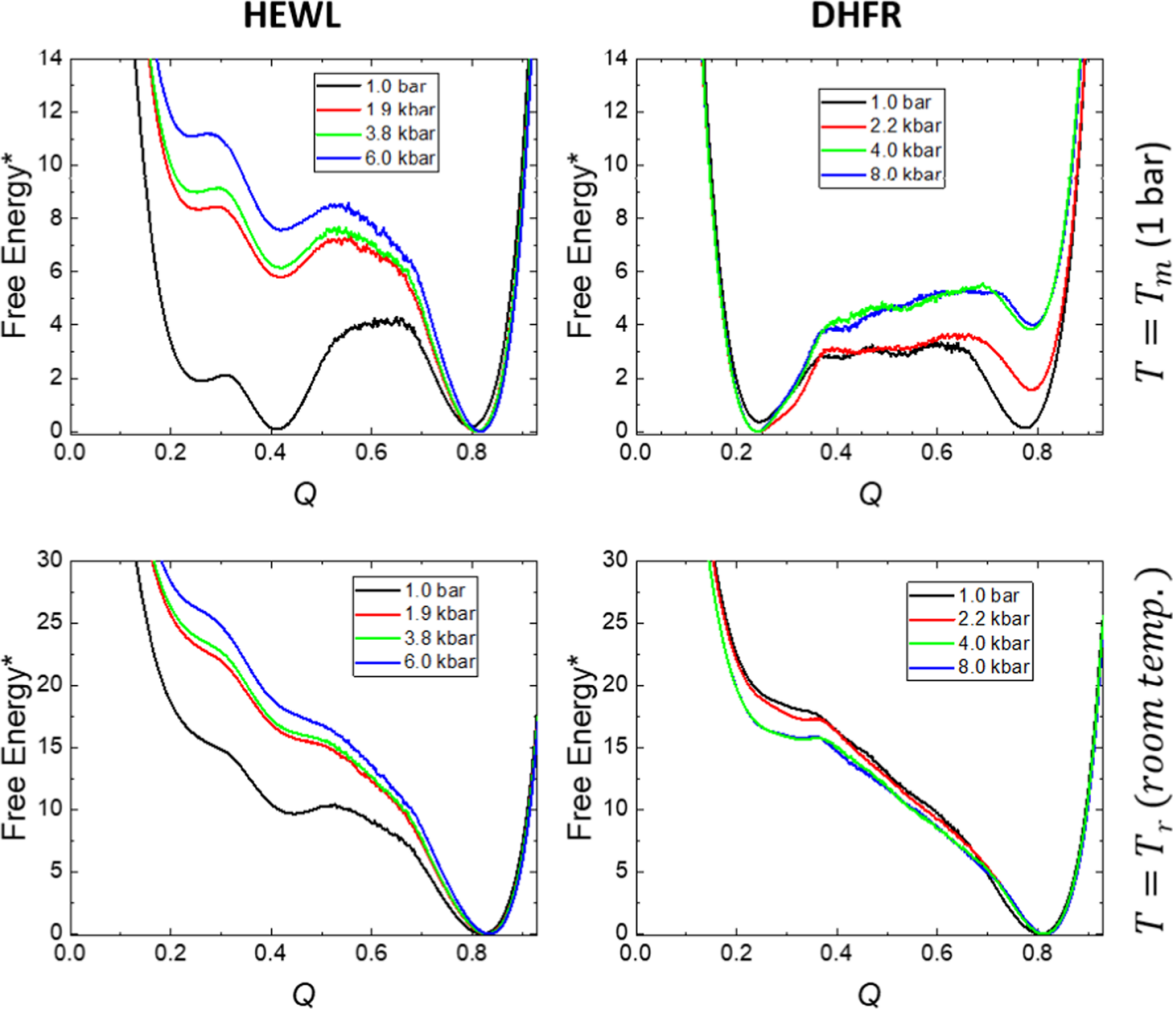
Free energy profiles (in reduced units) against the reaction coordinate *Q* (see eq 3 in the main text for details). Left column: results of HEWL. Right column: results of DHFR. The top graphs are computed at the transition temperature *T*_m_ corresponding to room pressure. The bottom graphs are computed at our approximation to room temperature, *T*_r_. The statistical error bars roughly correspond to the width of the drawn lines.

To better understand the thermodynamic features of the folding transition for these two proteins upon pressure changes, we have used the weighted histogram analysis method (WHAM)^70−72^ to obtain the free energy landscapes. This method uses information coming from all of the temperatures sampled in the parallel tempering procedure to define thermodynamic statistical weights for different states of the conformational space of the protein model simulated. Therefore, an estimation of the entropy is obtained, which combined with the energy provides the free energy. In Figure 4, we show the free energy profiles for HEWL (left graphs) and DHFR (right graphs) at every pressure and two different temperatures: the equilibrium temperature *T*_m_ computed at room pressure for each protein (upper graphs) and our estimation of room temperature, *T*_r_ (bottom graphs), which as mentioned in Section II is computed at 90% of the *T*_m_ values and will be used later for kinetic simulations. For the *x*-axes in these plots, we have used
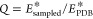
3where *E*_sampled_^*^ is the reduced energy of the sampled conformations of the model along the simulation and *E*_PDB_^*^ is the reduced energy of the experimental native conformation, according to our energy scale. The latter is just the total number of native contacts with a minus sign. This definition of *Q* roughly corresponds to the degree of “nativeness” of the conformations sampled along the simulations. We have previously used this ratio^67,73^ instead of the fraction of native contacts customarily used in structure-based models since it avoids an arbitrary definition of a threshold to determine whether a native contact is formed or not in a given conformation, something which may become controversial.^74^ With our scale, which can be considered as a reasonable folding coordinate for structure-based interaction models,^75^ we just obtain slightly lower values of *Q* for the folded state than with the standard native contact fraction, especially at *T*_m_, given the structural fluctuations present in the native state, which slightly reduce the energy in comparison with that computed for the experimental structure, *E*_PDB_^*^.

The free energies computed at *T*_m_ reflect the pressure effects commented above, with an increase in pressure further destabilizing the unfolded state in the case of HEWL (therefore, in relative terms, stabilizing the native state), with the opposite effect observed in DHFR. More interestingly, the profile for lysozyme at 1 bar shows two minima at lower *Q* values, signaling the presence of a thermodynamic intermediate in the folding process. The free energy barrier separating this intermediate from the unfolded state is clearly less than that separating the native state, and it becomes even lower as pressure increases. That is why the unfolded–intermediate transition can be effectively considered as barrierless at *T*_m_, which is the reason for the asymmetry in the heat capacity curve shown at room pressure in Figure 3a, which is hardly noticeable at higher pressures. The folding pathway of lysozyme at room pressure has been described as a complex one, involving intermediate states.^57,58^ With our model, an intermediate that is populated at low values of the folding coordinate has to be considered with some caution, at least from the structural point of view, since only native contacts are considered in our attractive potential, instead of the sequence-dependent interactions, which are more likely to stabilize non-native contacts in these conditions.^76^ That is why we have not pursued a more detailed structural analysis of this intermediate. However, the presence of a complex free energy landscape, which is pressure-dependent even though we have obtained it from very similar native structures, is worth mentioning.

For DHFR (right column plots in Figure 4), there are some local minima on top of a very broad barrier separating the folded from the unfolded states. However, the very small barriers existing among these minima, together with the high free energies they show, make their populations rather small and not relevant from a thermodynamic point of view.

When the temperature is reduced to room conditions (plots at the bottom row of Figure 4), the native state is the only one thermodynamically favored at any pressure. The free energy landscapes still show some roughness, apparently more important for HEWL at 1 bar than for the other pressures or for DHFR at any pressure. To better analyze the pressure effects in these conditions, we show in the next section the results of our kinetic analysis.

### IV.II Kinetic Simulations

**Figure 5:**
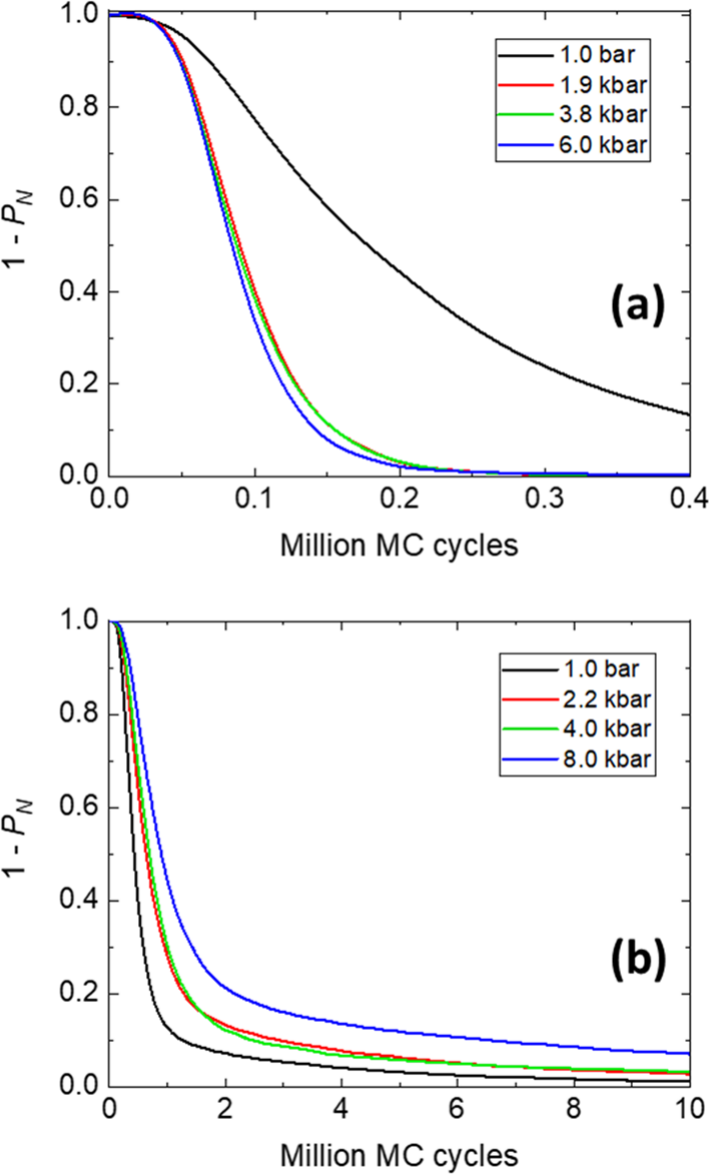
Kinetic results of (a) HEWL and (b) DHFR computed at room temperature. The graphs show the evolution of the population of the model chain from the unfolded state toward the native state.

For every protein and pressure conditions, we have started 10^4^ independent simulations at random conformations corresponding to the unfolded state and analyzed how long (in Monte Carlo cycles) it takes for every one of them to reach the native state, as described in Section II. This is a way of measuring the folding kinetics by exploring the behavior of a population of chains that, starting from the unfolded state, evolves toward the native state at room temperature *T*_r_, where this state is the only one thermodynamically stable. The results from these calculations are shown in Figure 5. In the *y*-axes, we have included the fraction of the original populations, which has not reached the native state after a given number of Monte Carlo cycles; that is why we have termed this fraction 1–*P*_N_, since *P*_N_ represents the fraction of the original population already folded, and we cannot claim, given the methodology employed, if the surviving population is still in the unfolded state or in possible kinetic intermediates appearing along the folding pathways.

Monte Carlo kinetics provides a rough description of the folding process kinetics, given the artificial nature of the conformational changes implemented in the sampling procedure. Even though the trajectories computed permit a more detailed numerical analysis, we have deliberately limited ourselves to global features, related to the final folding, which happens at a time scale orders of magnitude larger than the approximate scale of the local moves used in our kinetic simulations. Under these conditions, it is reasonable to assume a direct relation between the number of MC cycles and the real time, as we have done when analyzing the pressure effects in Figure 5.

By comparing the different scales in the horizontal axes for both proteins, HEWL in Figure 5a and DHFR in Figure 5b, our results show that at *T*_r_ the folding of the latter is much slower than the former, a fact that had been previously reported from experimental results,^77^ given the very slow folding of DHFR.^60,61,63,78,79^

For lysozyme, the kinetic results show an increase in the folding speed as pressure is increased, with small variations for the three high-pressure results. This is quite coincident with the free energy profiles at the same temperature for this protein shown in Figure 4 (bottom left graph). The kinetic results cannot be fitted to a single exponential decay (data not shown) at any pressure, as it would correspond to a single barrier transition from the unfolded to the folded state. As a matter of fact, kinetic models with one or two intermediates have to be used,^80^ showing that the kinetic folding pathway can become even more complicated than the transition analyzed from a thermodynamic point of view.

For DHFR, the kinetic curves show an initial fast evolution, followed by a very slow decay of the non-native population. Interestingly, kinetic experiments following DHFR refolding after urea unfolding have shown a relatively fast structural collapse (in about 300 μs) followed by a much slower folding step.^61,81^ For this protein, we had to use complex kinetic mechanisms with up to three intermediates to numerically fit the behavior shown in Figure 5b. The effect of pressure on DHFR is, once more, opposite to that shown in HEWL; now, an increase in pressure implies a slower folding process, with almost no difference between the results at 2.2 and 4.0 kbar. This is not the same behavior observed in the free energy profiles for DHFR at *T*_r_, the right lower panel of Figure 4.

**Figure 6:**
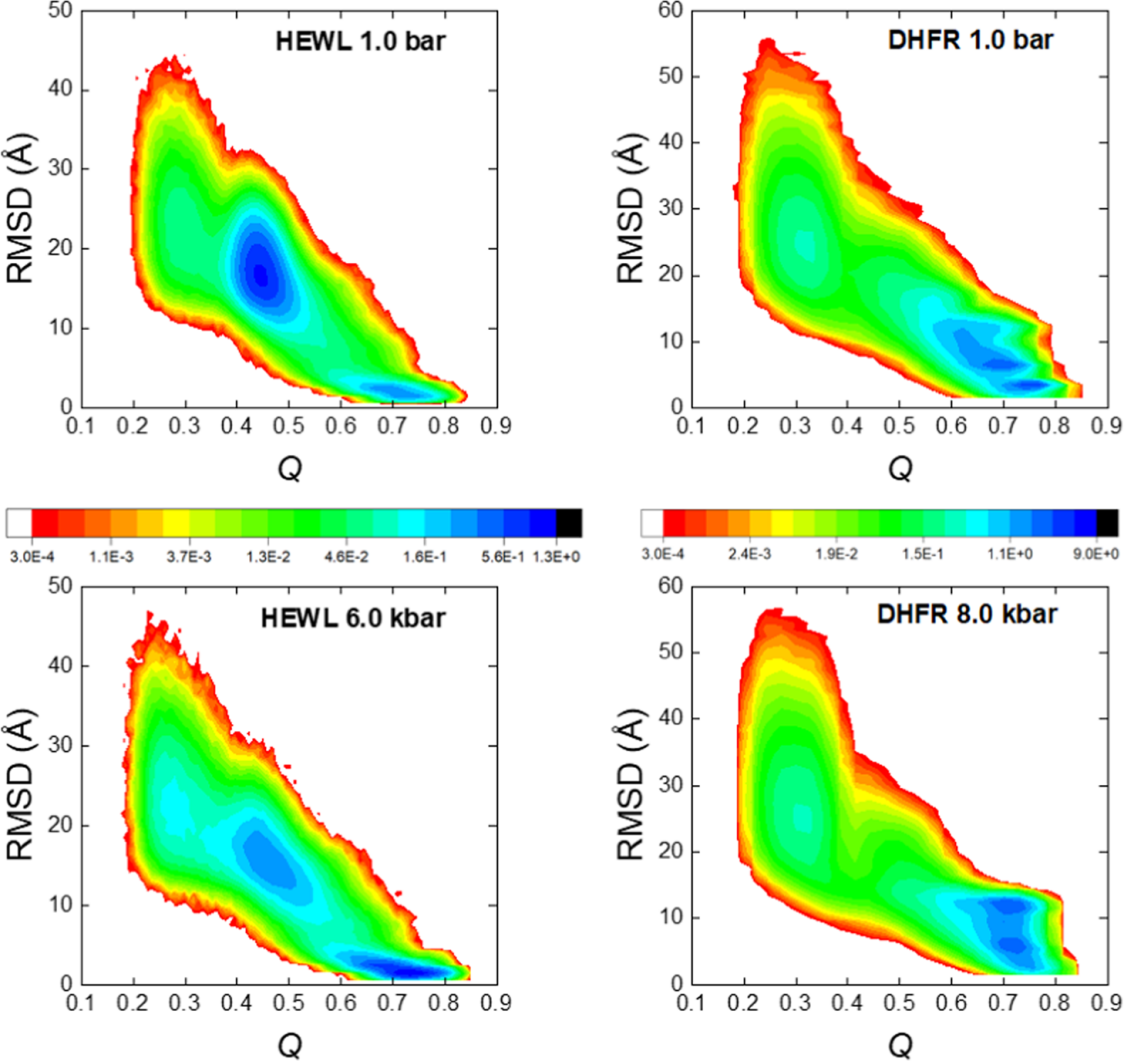
Populations sampled from the kinetic simulations at room temperature, *T*_r_. The color scales, as indicated in the rainbow panels, are percentage populations on a logarithmic scale. The RMSD values are referred to the experimental structure at the corresponding pressure. *Q* values are computed as defined in eq 3. Left column: results of HEWL. Right column: results of DHFR. The top graphs are computed at room pressure. The bottom graphs are computed at our highest pressure.

To try to better understand the kinetic behavior shown by our model for both proteins, we have analyzed the conformations sampled along the folding pathways. In Figure 6, we show the results obtained as two-dimensional histograms, where the relative populations of the sampled conformations are represented as a function of the folding coordinate *Q*, as defined in eq 3, and the structural RMSD between any sampled conformation and the corresponding experimental native structure at the level of α-carbons. In these histograms, we should recall that the population of the native state does not accumulate, even though it is the only one thermodynamically stable at room temperature, since the trajectories are stopped when this state is reached, according to the threshold described above.

For HEWL, we observe what can be considered as a rapid transition from the initial unfolded state, at *Q* ∼ 0.3, toward an intermediate state, at *Q* ∼ 0.45. This intermediate also appears in the thermodynamic free energy profiles shown in Figure 4. The population of this intermediate is high at 1 bar and is reduced at higher pressures (the behaviors of our intermediate pressures are very similar to those computed at 6.0 kbar shown in Figure 6). This eventually implies a faster transition toward the native state at high pressures, as shown in the kinetic curves depicted in Figure 5a.

For DHFR, there are not populated intermediates at low values of the folding coordinate *Q*, a situation that would favor the rapid initial trend of the kinetic pathway commented on above. However, we can observe several intermediates around *Q* ∼ 0.65–0.8, with barriers of various heights among them, as guessed from the reduction of the populations sampled at this region, which frame the different intermediates. This situation can be blamed for the very slow final evolution observed in our kinetic results for this protein. At room temperature, the thermal fluctuations are relatively small, and the time required to leave these local minima can be very large (therefore explaining their large populations).

## V Conclusions

In this work, we have used a simple structure-based model to study the pressure–temperature phase equilibrium of globular proteins, as well as the folding kinetics at different pressures, with molecular simulations. All of the pressure dependence of our interaction potential is based only on experimental structures previously determined at several pressures, for two test proteins: hen egg-white lysozyme (HEWL) and dihydrofolate reductase (DHFR). To this end, we have used a simple, coarse-grained representation of both the polypeptide chain geometry and the interactions that favor the energetic stability of the native state.

**Figure 7:**
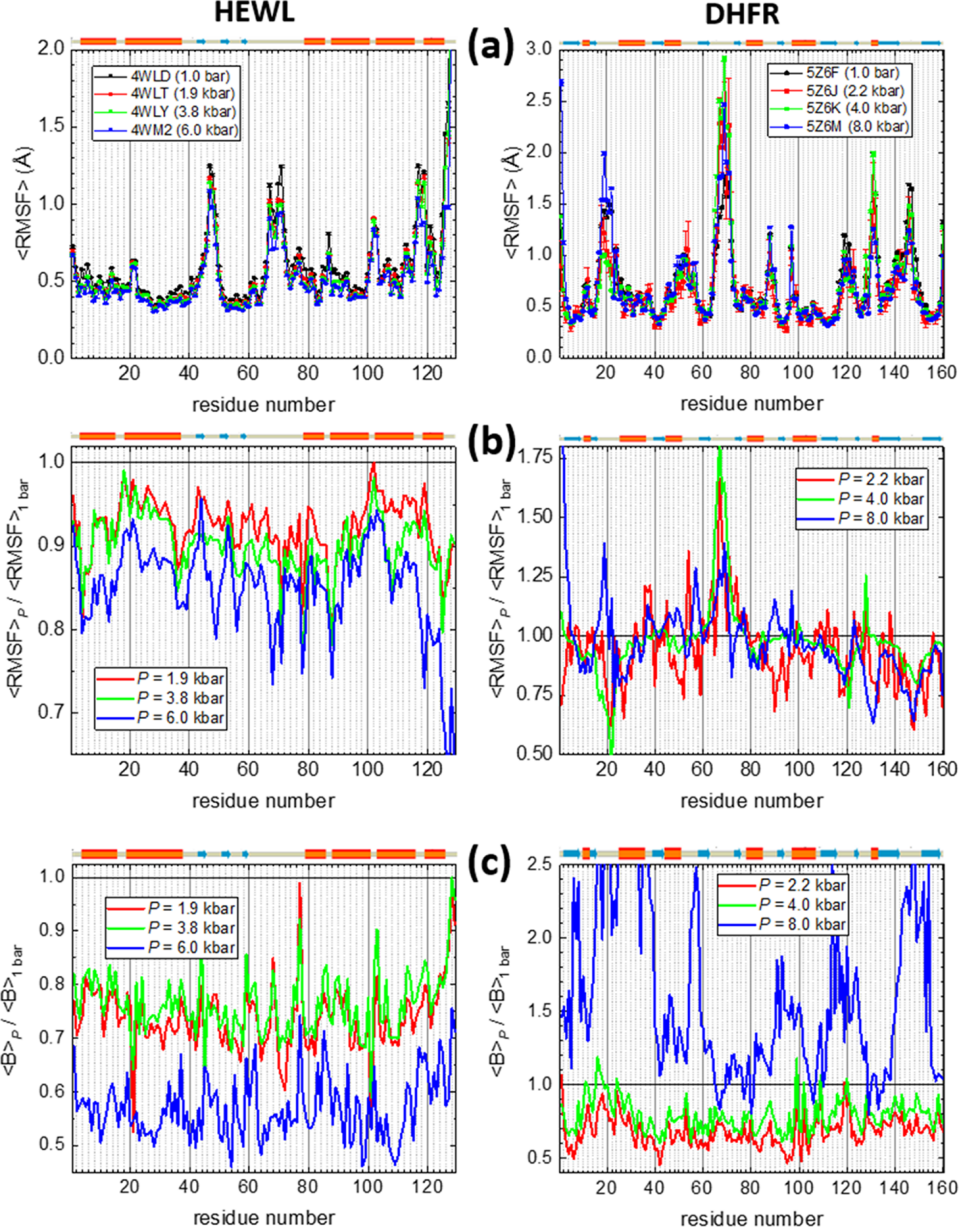
(a) Residue-level structural fluctuations for the native state simulated at room temperature (with respect to the X-ray native structures) and the indicated pressures for HEWL (left column) and DHFR (right column). The error bars are standard errors over the set of computed conformations. (b) Similar fluctuations as in (a), normalized with the corresponding fluctuations at room pressure. (c) Experimental B-factors, taken from the PDB files, averaged over the atoms in each residue and normalized with the values at room pressure.

An initial analysis of the pressure effects on the native structures, as discussed in the corresponding publications for HEWL^53^ and DHFR^54^ and summarized in Table 1 and Figure 2, could incorrectly indicate that these effects are too small to have any relevant consequence on the results, especially given the coarse-grained representation we are using. However, we have observed a thermal destabilization of the native state of DHFR and stabilization of HEWL as pressure increases. This corresponds to the two possible behaviors observed for the pressure–temperature phase diagrams of globular proteins,^27,28^ depending on the sign of the protein partial volume change upon unfolding. It is particularly interesting that, experimentally, this change depends on the cavities present in the native structure and on the possibility of water molecules being injected inside these cavities. Since the chain representation we are using only considers one spherical interaction site per residue (therefore, no real cavity analysis can be done at the level of the coarse-grained model) and the solvent is just implicitly considered, this could be taken as a surprising result. We have tried to rationalize it further by computing the structural fluctuations of the native state as represented by our coarse-grained model. In Figure 7, we show the fluctuations along the sequence for both proteins, simulated in equilibrium at room temperature, using the parallel tempering technique already described to warrant a proper equilibration. The graphs in row (a) correspond to the absolute values of the root-mean-square fluctuations (RMSFs) of each model site, relative to the α-carbons of the native conformation, averaged over the sampled conformations. To better appreciate the pressure effects, the graphs in row (b) show the ratios of the results at high pressure with respect to room pressure. For HEWL, fluctuations are less in the helical regions and are monotonically reduced as pressure increases. This is explained by the consistently larger number of native contacts found at larger pressures, scattered all around the contact map, as shown in Figure 2a, and partially justifies the equivalently larger transition temperatures for this protein collected in Figure 3c. In DHFR, on the other hand, the results in Figure 7 are much more complicated. The fluctuation amplitudes are different (with ratios smaller or larger than 1) along the protein sequence, and the same can be said about the trend with pressure itself. This means that pressure may compress some cavities, but others may expand as well if water molecules are injected inside them, as shown in the analysis of the experimental structures.^54^ This behavior is clearly different from the one we have obtained for lysozyme, and therefore, the thermodynamic effect of pressure on the transition is also different. These results indicate that a global measurement of the structural difference among structures, as the results for the global RMSD in Table 1, can indeed not be enough to properly appreciate the effects of pressure, either in the real native structures or in their coarse-grained representations.

To complete this analysis, in Figure 7c, we show the experimental B-factors taken from the crystallographic data in the PDB files.^53,54^ The results shown correspond to average values over the atoms in each residue, divided by the corresponding values at 1 bar. B-factors provide an estimation of the thermal fluctuations for the different atoms in the crystal structure and are therefore an experimental analogue, with the restrictions of the crystal packing, of the RMSF values we have computed in rows (a) and (b). We have checked that using the B-factors of the α-carbons alone produces equivalent pressure dependences of the ratios plotted.

The results of lysozyme are qualitatively similar to the computed structural fluctuations in Figure 7b, although for the B-factors there is a larger similarity in the ratios at intermediate pressures and a larger difference with the results at 6 kbar than with the <RMSF> results. This kind of global behavior is quite similar to what we have obtained for the heat capacity curves (shown in Figure 3a) or the free energy profiles (Figure 4) of HEWL and can be therefore considered as further validation of our simulation results.

For DHFR, the results for the B-factors are again rather complex. Actually, for the highest pressure the fluctuations are huge. The researchers who measured the experimental structures for this protein reported^54^ that at slightly higher pressures the X-ray reflections disappear, probably indicating the pressure denaturation of the protein, and that at 8 kbar the resolution is less than that at the other pressures. At intermediate pressures, there is not a clear trend in the B-factor ratios, as it also happens for <RMSF> in this protein, with values usually less than 1 but in some positions also larger than 1. The lack of ligands in our coarse-grained representation prevents a better comparison with the experimental fluctuations for this protein.

The approximate kinetic results we have obtained from the model at room temperature also show a clear pressure dependence, as shown in Figures 5 and 6. Of course, more detailed structural analyses can be performed on these (as well as the thermodynamic) data. However, we want to recall that our model lacks some important features of the real proteins considered, namely the sulfur bridges in the case of HEWL and the ligands in the case of DHFR. Since these groups create specific interactions that surely affect the folding/unfolding process, in this manuscript, we have limited our comparison between simulation results and experimental evidence to very global characteristics, as mentioned in Section IV. Even with these restrictions, which could be partially avoided using more complex models (an aim for future work), this comparison quite supports the validity of our goals.

This way, we have proved that the minute differences shown by the experimental native conformations when pressure is increased are indeed reflected in reasonable pressure-dependent thermodynamic and kinetic behaviors for the corresponding folding processes. If the number of structures determined at high pressures continues to increase, as it has started to be the case in recent years, the approach described in this work can be a useful tool in the analysis of these pressure effects, and we hope it can be extended to different implementations of structure-based models and sampling techniques.

## References

[ref1] Piana S.; Lindorff-Larsen K.; Shaw D. E. Protein Folding Kinetics and Thermodynamics from Atomistic Simulation. Proc. Natl. Acad. Sci. U.S.A. 2012, 109, 17845–17850. 10.1073/pnas.1201811109. 22822217 PMC3497772

[ref2] Tozzini V. Coarse-Grained Models for Proteins. Curr. Opin. Struct. Biol. 2005, 15, 144–150. 10.1016/j.sbi.2005.02.005. 15837171

[ref3] Kmiecik S.; Gront D.; Kolinski M.; Wieteska L.; Dawid A. E.; Kolinski A. Coarse-Grained Protein Models and Their Applications. Chem. Rev. 2016, 116, 7898–7936. 10.1021/acs.chemrev.6b00163. 27333362

[ref4] Ingólfsson H. I.; Lopez C. A.; Uusitalo J. J.; de Jong D. H.; Gopal S. M.; Periole X.; Marrink S. J. The Power of Coarse Graining in Biomolecular Simulations. Wiley Interdiscip. Rev. Comput. Mol. Sci. 2014, 4, 225–248. 10.1002/wcms.1169. 25309628 PMC4171755

[ref5] Blaszczyk M.; Gront D.; Kmiecik S.; Kurcinski M.; Kolinski M.; Ciemny M. P.; Ziolkowska K.; Panek M.; Kolinski A. Protein Structure Prediction Using Coarse-Grained Models. In Computational Methods to Study the Structure and Dynamics of Biomolecules and Biomolecular Processes;Liwo A., Ed.; Springer International Publishing: Cham, 2019; pp 27–59.

[ref6] Takada S. Go Model Revisited. Biophys. Physicobiol. 2019, 16, 248–255. 10.2142/biophysico.16.0_248. 31984178 PMC6976017

[ref7] Nymeyer H.; Garcia A. E.; Onuchic J. N. Folding Funnels and Frustration in Off-Lattice Minimalist Protein Landscapes. Proc. Natl. Acad. Sci. U.S.A. 1998, 95, 5921–5928. 10.1073/pnas.95.11.5921. 9600893 PMC34496

[ref8] Onuchic J. N.; Luthey-Schulten Z.; Wolynes P. G. Theory of Protein Folding: The Energy Landscape Perspective. Annu. Rev. Phys. Chem. 1997, 48, 545–600. 10.1146/annurev.physchem.48.1.545. 9348663

[ref9] Wolynes P. G.; Eaton W. A.; Fersht A. R. Chemical Physics of Protein Folding. Proc. Natl. Acad. Sci. U.S.A. 2012, 109, 17770–17771. 10.1073/pnas.1215733109. 23112193 PMC3497751

[ref10] Jana B.; Morcos F.; Onuchic J. N. From Structure to Function: The Convergence of Structure Based Models and Co-Evolutionary Information. Phys. Chem. Chem. Phys. 2014, 16, 6496–6507. 10.1039/C3CP55275F. 24603809

[ref11] Estacio S. G.; Fernandes C. S.; Krobath H.; Faisca P. F.; Shakhnovich E. I. Robustness of Atomistic Go Models in Predicting Native-Like Folding Intermediates. J. Chem. Phys. 2012, 137, 085102 10.1063/1.4747492. 22938266

[ref12] Paci E.; Vendruscolo M.; Karplus M. Validity of Go Models: Comparison with a Solvent-Shielded Empirical Energy Decomposition. Biophys. J. 2002, 83, 3032–3038. 10.1016/S0006-3495(02)75308-3. 12496075 PMC1302383

[ref13] Larriva M.; Prieto L.; Bruscolini P.; Rey A. A Simple Simulation Model Can Reproduce the Thermodynamic Folding Intermediate of Apoflavodoxin. Proteins 2010, 78, 73–82. 10.1002/prot.22521. 19688823

[ref14] Chen T.; Chan H. S. Native Contact Density and Nonnative Hydrophobic Effects in the Folding of Bacterial Immunity Proteins. PLoS Comput. Biol. 2015, 11, e1004260 10.1371/journal.pcbi.1004260. 26016652 PMC4446218

[ref15] Ruiz-Ortiz I.; De Sancho D. Competitive Binding of Hif-1alpha and Cited2 to the Taz1 Domain of Cbp from Molecular Simulations. Phys. Chem. Chem. Phys. 2020, 22, 8118–8127. 10.1039/D0CP00328J. 32242581

[ref16] Clementi C.; Nymeyer H.; Onuchic J. N. Topological and Energetic Factors: What Determines the Structural Details of the Transition State Ensemble and ″En-Route″ Intermediates for Protein Folding? An Investigation for Small Globular Proteins. J. Mol. Biol. 2000, 298, 937–953. 10.1006/jmbi.2000.3693. 10801360

[ref17] Plotkin S. S. Speeding Protein Folding Beyond the G(O) Model: How a Little Frustration Sometimes Helps. Proteins 2001, 45, 337–345. 10.1002/prot.1154. 11746681

[ref18] Balny C.; Masson P.; Heremans K. High Pressure Effects on Biological Macromolecules: From Structural Changes to Alteration of Cellular Processes. Biochim. Biophys. Acta, Protein Struct. Mol. Enzymol. 2002, 1595, 3–10. 10.1016/S0167-4838(01)00331-4. 11983383

[ref19] Chalikian T. V.; Macgregor R. B. Jr. Origins of Pressure-Induced Protein Transitions. J. Mol. Biol. 2009, 394, 834–842. 10.1016/j.jmb.2009.10.020. 19837081

[ref20] Chen C. R.; Makhatadze G. I. Molecular Determinant of the Effects of Hydrostatic Pressure on Protein Folding Stability. Nat. Commun. 2017, 8, 14561 10.1038/ncomms14561. 28169271 PMC5309723

[ref21] Cheung J. K.; Shah P.; Truskett T. M. Heteropolymer Collapse Theory for Protein Folding in the Pressure-Temperature Plane. Biophys. J. 2006, 91, 2427–2435. 10.1529/biophysj.106.081802. 16844760 PMC1562399

[ref22] Galazka V. B.; Dickinson E.; Ledward D. A. Influence of High Pressure Processing on Protein Solutions and Emulsions. Curr. Opin. Colloid Interface Sci. 2000, 5, 182–187. 10.1016/S1359-0294(00)00055-8.

[ref23] Grigera J. R.; McCarthy A. N. The Behavior of the Hydrophobic Effect under Pressure and Protein Denaturation. Biophys. J. 2010, 98, 1626–1631. 10.1016/j.bpj.2009.12.4298. 20409483 PMC2856145

[ref24] Silva J. L.; Foguel D. Hydration, Cavities and Volume in Protein Folding, Aggregation and Amyloid Assembly. Phys. Biol. 2009, 6, 015002 10.1088/1478-3975/6/1/015002. 19208935

[ref25] Silva J. L.; Foguel D.; Royer C. A. Pressure Provides New Insights into Protein Folding, Dynamics and Structure. Trends Biochem. Sci. 2001, 26, 612–618. 10.1016/S0968-0004(01)01949-1. 11590014

[ref26] Winter R.; Dzwolak W. Exploring the Temperature-Pressure Configurational Landscape of Biomolecules: From Lipid Membranes to Proteins. Philos. Trans. R. Soc., A 2005, 363, 537–562. 10.1098/rsta.2004.1507. 15664898

[ref27] Roche J.; Caro J. A.; Norberto D. R.; Barthe P.; Roumestand C.; Schlessman J. L.; Garcia A. E.; Garcia-Moreno B. E.; Royer C. A. Cavities Determine the Pressure Unfolding of Proteins. Proc. Natl. Acad. Sci. U.S.A. 2012, 109, 6945–6950. 10.1073/pnas.1200915109. 22496593 PMC3344970

[ref28] Roche J.; Dellarole M.; Caro J. A.; Norberto D. R.; Garcia A. E.; Garcia-Moreno B.; Roumestand C.; Royer C. A. Effect of Internal Cavities on Folding Rates and Routes Revealed by Real-Time Pressure-Jump Nmr Spectroscopy. J. Am. Chem. Soc. 2013, 135, 14610–14618. 10.1021/ja406682e. 23987660

[ref29] Ghosh T.; Garcia A. E.; Garde S. Molecular Dynamics Simulations of Pressure Effects on Hydrophobic Interactions. J. Am. Chem. Soc. 2001, 123, 10997–11003. 10.1021/ja010446v. 11686704

[ref30] Dias C. L.; Chan H. S. Pressure-Dependent Properties of Elementary Hydrophobic Interactions: Ramifications for Activation Properties of Protein Folding. J. Phys. Chem. B 2014, 118, 7488–7509. 10.1021/jp501935f. 24933471

[ref31] Hillson N.; Onuchic J. N.; Garcia A. E. Pressure-Induced Protein-Folding/Unfolding Kinetics. Proc. Natl. Acad. Sci. U.S.A. 1999, 96, 14848–14853. 10.1073/pnas.96.26.14848. 10611301 PMC24736

[ref32] Gasic A. G.; Cheung M. S. A Tale of Two Desolvation Potentials: An Investigation of Protein Behavior under High Hydrostatic Pressure. J. Phys. Chem. B 2020, 124, 1619–1627. 10.1021/acs.jpcb.9b10734. 32026686 PMC8691387

[ref33] Perezzan R.; Rey A. Simulating Protein Unfolding under Pressure with a Coarse-Grained Model. J. Chem. Phys. 2012, 137, 185102 10.1063/1.4765057. 23163394

[ref34] Onuchic J. N.; Nymeyer H.; Garcia A. E.; Chahine J.; Socci N. D. The Energy Landscape Theory of Protein Folding: Insights into Folding Mechanisms and Scenarios. Adv. Protein Chem. 2000, 53, 87–152. 10.1016/S0065-3233(00)53003-4. 10751944

[ref35] Whitford P. C.; Noel J. K.; Gosavi S.; Schug A.; Sanbonmatsu K. Y.; Onuchic J. N. An All-Atom Structure-Based Potential for Proteins: Bridging Minimal Models with All-Atom Empirical Forcefields. Proteins 2009, 75, 430–441. 10.1002/prot.22253. 18837035 PMC3439813

[ref36] Gallo P.; Amann-Winkel K.; Angell C. A.; Anisimov M. A.; Caupin F.; Chakravarty C.; Lascaris E.; Loerting T.; Panagiotopoulos A. Z.; Russo J.; et al. Water: A Tale of Two Liquids. Chem. Rev. 2016, 116, 7463–7500. 10.1021/acs.chemrev.5b00750. 27380438 PMC5424717

[ref37] Kim S. B.; Palmer J. C.; Debenedetti P. G. Computational Investigation of Cold Denaturation in the Trp-Cage Miniprotein. Proc. Natl. Acad. Sci. U.S.A. 2016, 113, 8991–8996. 10.1073/pnas.1607500113. 27457961 PMC4987839

[ref38] Prieto L.; de Sancho D.; Rey A. Thermodynamics of Go-Type Models for Protein Folding. J. Chem. Phys. 2005, 123, 154903 10.1063/1.2064888. 16252968

[ref39] Prieto L.; Rey A. Influence of the Native Topology on the Folding Barrier for Small Proteins. J. Chem. Phys. 2007, 127, 175101 10.1063/1.2780154. 17994851

[ref40] Fukunishi H.; Watanabe O.; Takada S. On the Hamiltonian Replica Exchange Method for Efficient Sampling of Biomolecular Systems: Application to Protein Structure Prediction. J. Chem. Phys. 2002, 116, 9058–9067. 10.1063/1.1472510.

[ref41] Sugita Y.; Okamoto Y. Replica-Exchange Molecular Dynamics Method for Protein Folding. Chem. Phys. Lett. 1999, 314, 141–151. 10.1016/S0009-2614(99)01123-9.

[ref42] Berman H. M.; Battistuz T.; Bhat T. N.; Bluhm W. F.; Bourne P. E.; Burkhardt K.; Feng Z.; Gilliland G. L.; Iype L.; Jain S.; et al. The Protein Data Bank. Acta Crystallogr., Sect. D: Biol. Crystallogr. 2002, 58, 899–907. 10.1107/S0907444902003451. 12037327

[ref43] Enciso M.; Rey A. Improvement of Structure-Based Potentials for Protein Folding by Native and Nonnative Hydrogen Bonds. Biophys. J. 2011, 101, 1474–1482. 10.1016/j.bpj.2011.08.017. 21943429 PMC3177075

[ref44] Soler M. A.; Rey A.; Faisca P. F. Steric Confinement and Enhanced Local Flexibility Assist Knotting in Simple Models of Protein Folding. Phys. Chem. Chem. Phys. 2016, 18, 26391–26403. 10.1039/C6CP05086G. 27722468

[ref45] Faísca P. F.; Travasso R. D.; Parisi A.; Rey A. Why Do Protein Folding Rates Correlate with Metrics of Native Topology?. PLoS One 2012, 7, e35599 10.1371/journal.pone.0035599. 22558173 PMC3338708

[ref46] Krobath H.; Rey A.; Faisca P. F. How Determinant Is N-Terminal to C-Terminal Coupling for Protein Folding?. Phys. Chem. Chem. Phys. 2015, 17, 3512–3524. 10.1039/C4CP05178E. 25536450

[ref47] Akasaka K.; Kitahara R.; Kamatari Y. O. Exploring the Folding Energy Landscape with Pressure. Arch. Biochem. Biophys. 2013, 531, 110–115. 10.1016/j.abb.2012.11.016. 23246376

[ref48] Dias C. L. Unifying Microscopic Mechanism for Pressure and Cold Denaturations of Proteins. Phys. Rev. Lett. 2012, 109, 048104 10.1103/PhysRevLett.109.048104. 23006112

[ref49] Harano Y.; Yoshidome T.; Kinoshita M. Molecular Mechanism of Pressure Denaturation of Proteins. J. Chem. Phys. 2008, 129, 145103 10.1063/1.2991176. 19045168

[ref50] Marchal S.; Torrent J.; Masson P.; Kornblatt J. M.; Tortora P.; Fusi P.; Lange R.; Balny C. The Powerful High Pressure Tool for Protein Conformational Studies. Braz. J. Med. Biol. Res. 2005, 38, 1175–1183. 10.1590/S0100-879X2005000800004. 16082457

[ref51] Royer C. A. Insights into the Role of Hydration in Protein Structure and Stability Obtained through Hydrostatic Pressure Studies. Braz. J. Med. Biol. Res. 2005, 38, 1167–1173. 10.1590/S0100-879X2005000800003. 16082456

[ref52] Katrusiak A.; Dauter Z. Compressibility of Lysozyme Protein Crystals by X-Ray Diffraction. Acta Crystallogr., Sect. D: Biol. Crystallogr. 1996, 52, 607–608. 10.1107/S0907444996000431. 15299694

[ref53] Yamada H.; Nagae T.; Watanabe N. High-Pressure Protein Crystallography of Hen Egg-White Lysozyme. Acta Crystallogr., Sect. D: Biol. Crystallogr. 2015, 71, 742–753. 10.1107/S1399004715000292. 25849385 PMC4388261

[ref54] Nagae T.; Yamada H.; Watanabe N. High-Pressure Protein Crystal Structure Analysis of *Escherichia coli* Dihydrofolate Reductase Complexed with Folate and Nadp(). Acta Crystallogr., Sect. D: Struct. Biol. 2018, 74, 895–905. 10.1107/S2059798318009397. 30198899 PMC6130465

[ref55] Calandrini V.; Kneller G. R. Influence of Pressure on the Slow and Fast Fractional Relaxation Dynamics in Lysozyme: A Simulation Study. J. Chem. Phys. 2008, 128, 065102 10.1063/1.2828769. 18282073

[ref56] Hedoux A.; Guinet Y.; Paccou L. Analysis of the Mechanism of Lysozyme Pressure Denaturation from Raman Spectroscopy Investigations, and Comparison with Thermal Denaturation. J. Phys. Chem. B 2011, 115, 6740–6748. 10.1021/jp2014836. 21542584

[ref57] Matagne A.; Dobson C. M. The Folding Process of Hen Lysozyme: A Perspective from the ’New View’. Cell. Mol. Life Sci. 1998, 54, 363–371. 10.1007/s000180050165. 9614974 PMC11147319

[ref58] Merlini G.; Bellotti V. Lysozyme: A Paradigmatic Molecule for the Investigation of Protein Structure, Function and Misfolding. Clin. Chim. Acta 2005, 357, 168–172. 10.1016/j.cccn.2005.03.022. 15913589

[ref59] Sasahara K.; Sakurai M.; Nitta K. Pressure Effect on Denaturant-Induced Unfolding of Hen Egg White Lysozyme. Proteins 2001, 44, 180–187. 10.1002/prot.1083. 11455591

[ref60] Clementi C.; Jennings P. A.; Onuchic J. N. How Native-State Topology Affects the Folding of Dihydrofolate Reductase and Interleukin-1beta. Proc. Natl. Acad. Sci. U.S.A. 2000, 97, 5871–5876. 10.1073/pnas.100547897. 10811910 PMC18526

[ref61] Heidary D. K.; O’Neill J. C. Jr.; Roy M.; Jennings P. A. An Essential Intermediate in the Folding of Dihydrofolate Reductase. Proc. Natl. Acad. Sci. U.S.A. 2000, 97, 5866–5870. 10.1073/pnas.100547697. 10811909 PMC18525

[ref62] Huang Q.; Rodgers J. M.; Hemley R. J.; Ichiye T. Effects of Pressure and Temperature on the Atomic Fluctuations of Dihydrofolate Reductase from a Psychropiezophile and a Mesophile. Int. J. Mol. Sci. 2019, 20, 1452 10.3390/ijms20061452. PMC6470811 30909394

[ref63] Ohmae E.; Kurumiya T.; Makino S.; Gekko K. Acid and Thermal Unfolding of *Escherichia coli* Dihydrofolate Reductase. J. Biochem. 1996, 120, 946–953. 10.1093/oxfordjournals.jbchem.a021511. 8982861

[ref64] Teixeira S. C. M.; Penhallurick R.; Hoopes J. T.; Hemley R. J.; Ichiye T. High-Pressure Structural Studies of Dihydrofolate Reductase in Solution. Biophys. J. 2019, 116, 336a 10.1016/j.bpj.2018.11.1831.

[ref65] Refaee M.; Tezuka T.; Akasaka K.; Williamson M. P. Pressure-Dependent Changes in the Solution Structure of Hen Egg-White Lysozyme. J. Mol. Biol. 2003, 327, 857–865. 10.1016/S0022-2836(03)00209-2. 12654268

[ref66] Rey-Stolle M. F.; Enciso M.; Rey A. Topology-Based Models and Nmr Structures in Protein Folding Simulations. J. Comput. Chem. 2009, 30, 1212–1219. 10.1002/jcc.21149. 18988253

[ref67] Rubio A. M.; Rey A. Design of a Structure-Based Model for Protein Folding from Flexible Conformations. Phys. Chem. Chem. Phys. 2019, 21, 6544–6552. 10.1039/C9CP00168A. 30848270

[ref68] Obuchi K.; Yamanobe T. Differential Scanning Calorimetry of Proteins under High Pressure. In Trends in High Pressure Bioscience and Biotechnology, Proceedings First International Conference on High Pressure Bioscience and Biotechnology; Hayashi R., Ed.; Elsevier, 2002; Vol. 19, pp 599–606.

[ref69] Yegorov A. Y.; Potekhin S. A. Lysozyme Stabilization under High Pressure: Differential Scanning Microcalorimetry. Mol. Biol. 2018, 52, 36–42. 10.1134/S0026893318010028. 29512634

[ref70] Ferrenberg A. M.; Swendsen R. H. Optimized Monte Carlo Data Analysis. Phys. Rev. Lett. 1989, 63, 1195–1198. 10.1103/PhysRevLett.63.1195. 10040500

[ref71] Kumar S.; Rosenberg J. M.; Bouzida D.; Swendsen R. H.; Kollman P. A. The Weighted Histogram Analysis Method for Free-Energy Calculations on Biomolecules. I. The Method. J. Comput. Chem. 1992, 13, 1011–1021. 10.1002/jcc.540130812.

[ref72] Chodera J. D.; Swope W. C.; Pitera J. W.; Seok C.; Dill K. A. Use of the Weighted Histogram Analysis Method for the Analysis of Simulated and Parallel Tempering Simulations. J. Chem. Theory Comput. 2007, 3, 26–41. 10.1021/ct0502864. 26627148

[ref73] Especial J.; Nunes A.; Rey A.; Faisca P. F. Hydrophobic Confinement Modulates Thermal Stability and Assists Knotting in the Folding of Tangled Proteins. Phys. Chem. Chem. Phys. 2019, 21, 11764–11775. 10.1039/C9CP01701A. 31114834

[ref74] Wołek K.; Cieplak M. Criteria for Folding in Structure-Based Models of Proteins. J. Chem. Phys. 2016, 144, 185102 10.1063/1.4948783. 27179507

[ref75] Best R. B.; Hummer G. Reaction Coordinates and Rates from Transition Paths. Proc. Natl. Acad. Sci. U.S.A. 2005, 102, 6732–6737. 10.1073/pnas.0408098102. 15814618 PMC1100744

[ref76] Paci E.; Vendruscolo M. Detection of Non-Native Hydrophobic Interactions in the Denatured State of Lysozyme by Molecular Dynamics Simulations. J. Phys.: Condens. Matter 2005, 17, S1617–S1626. 10.1088/0953-8984/17/18/017.

[ref77] Huang K. Kinetics of Protein Folding. In Lectures on Statistical Physics and Protein Folding; World Scientific, 2005; pp 95–103.

[ref78] Arai M.; Kataoka M.; Kuwajima K.; Matthews C. R.; Iwakura M. Effects of the Difference in the Unfolded-State Ensemble on the Folding of *Escherichia coli* Dihydrofolate Reductase. J. Mol. Biol. 2003, 329, 779–791. 10.1016/S0022-2836(03)00511-4. 12787677

[ref79] Arai M.; Maki K.; Takahashi H.; Iwakura M. Testing the Relationship between Foldability and the Early Folding Events of Dihydrofolate Reductase from *Escherichia coli*. J. Mol. Biol. 2003, 328, 273–288. 10.1016/S0022-2836(03)00212-2. 12684013

[ref80] Nolting B. Protein Folding Kinetics; Springer-Verlag: Berlin Heidelberg, 2006.

[ref81] Arai M.; Kondrashkina E.; Kayatekin C.; Matthews C. R.; Iwakura M.; Bilsel O. Microsecond Hydrophobic Collapse in the Folding of *Escherichia coli* Dihydrofolate Reductase, an Alpha/Beta-Type Protein. J. Mol. Biol. 2007, 368, 219–229. 10.1016/j.jmb.2007.01.085. 17331539

